# Paeonol Protects Rat Heart by Improving Regional Blood Perfusion during No-Reflow

**DOI:** 10.3389/fphys.2016.00298

**Published:** 2016-07-21

**Authors:** Lina Ma, Chia-Chen Chuang, Weiliang Weng, Le Zhao, Yongqiu Zheng, Jinyan Zhang, Li Zuo

**Affiliations:** ^1^Graduate School, Beijing University of Chinese MedicineBeijing, China; ^2^Institute of Basic Medical Sciences, Xiyuan Hospital, China Academy of Chinese Medical SciencesBeijing, China; ^3^Radiologic Sciences and Respiratory Therapy Division, School of Health and Rehabilitation Sciences, The Ohio State University College of MedicineColumbus, OH, USA; ^4^Interdisciplinary Biophysics Graduate Program, The Ohio State UniversityColumbus, OH, USA

**Keywords:** paeonol, myocardial injury, no-reflow, infarct size, rat

## Abstract

No-reflow phenomenon, defined as inadequate perfusion of myocardium without evident artery obstruction, occurs at a high incidence after coronary revascularization. The mechanisms underlying no-reflow is only partially understood. It is commonly caused by the swelling of endothelial cells, neutrophil accumulation, and vasoconstriction, which are all related to acute inflammation. Persistent no-reflow can lead to hospitalization and mortality. However, an effective preventive intervention has not yet been established. We have previously found that paeonol, an active extraction from the root of *Paeonia suffruticosa*, can benefit the heart function by inhibiting tissue damage after ischemia, reducing inflammation, and inducing vasodilatation. To further investigate the potential cardioprotective action of paeonol on no-reflow, healthy male Wistar rats were randomly divided into four groups: sham, ischemia-reperfusion (I/R) injury (left anterior descending coronary artery was ligated for 4 h followed by reperfusion for 8 h), and I/R injury pretreated with paeonol at two different doses. Real-time myocardial contrast echocardiography was used to monitor regional blood perfusion and cardiac functions. Our data indicated that paeonol treatment significantly reduces myocardial infarct area and no-reflow area (*n* = 8; *p* < 0.05). Regional myocardial perfusion (A·β) and cardiac functions such as ejection fraction, stroke volume, and fractional shortening were elevated by paeonol (*n* = 8; *p* < 0.05). Paeonol also lowered the serum levels of lactate dehydrogenase, creatine kinase, cardiac troponin T, and C-reactive protein, as indices of myocardial injury. Paeonol exerts beneficial effects on attenuating I/R-associated no-reflow injuries, and may be considered as a potential preventive treatment for cardiac diseases or post-coronary revascularization in which no-reflow often occurs.

## Introduction

Ischemic heart disease is a major cause of morbidity and mortality worldwide (Zuo et al., 2009, 2013; Zhu and Zuo, 2013; Sivaraman and Yellon, 2014). Coronary revascularization, including thrombolysis, percutaneous coronary intervention (PCI), and bypass grafting, is recognized as the most effective therapeutic strategy for coronary artery diseases (Niccoli et al., 2009; Serruys et al., 2009). However, a subset of patients continues to suffer from severe myocardial ischemia caused by factors such as myocardial interstitial edema, microvascular spasm, endothelial cell injury/swelling, neutrophil accumulation, micro-thromboembolism, and vasoconstriction related to acute inflammation (Rezkalla and Kloner, 2002; Kaul, 2014; Shao et al., 2014). The persistence of these symptoms, termed no-reflow phenomena, can cause insufficient regional blood perfusion, left ventricular (LV) dysfunction, and even cardiac death (Kawano et al., 2005; Chan et al., 2012; Zeng et al., 2012). Several attempts have been made to resolve no-reflow phenomenon with novel interventions associated with Western medicine, yet no ideal strategy has been established (Ramjane et al., 2008; Salinas et al., 2012).

Approaches that use traditional Chinese medicinal plants and their active ingredients, such as Tongxinluo capsule, have been emerging due to their multi-targeted pharmacological effects during no-reflow treatment (Wu et al., 2006; Liu et al., 2011). Other Chinese medicines, such as paeonol, a main constituent extracted from the root of *Paeonia suffruticosa*, is regarded as a traditional Chinese herb that belongs to the Ranunculaceae family. Studies have observed that paeonol exhibits a wide range of biological effects, including anti-inflammatory, immune regulatory, anti-tumor, and anti-oxidative effects (Sun et al., 2008; Jin et al., 2016). Our previous studies on rat models have demonstrated that paeonol is therapeutically beneficial for myocardial infarction by inhibiting tissue damage after ischemia and inducing vasodilatation in the mesenteric artery *in vivo* (Li Y.K. et al., 2010; Zhang et al., 2013). Other studies showed that paeonol can reduce myocardial damage by preventing apoptosis *in vivo* (Nizamutdinova et al., 2008). However, our understanding of paeonol as a cardioprotective agent against myocardial ischemia/reperfusion (I/R)-associated no-reflow phenomenon, is still largely unknown. Therefore, the current study particularly aims to investigate the effect of paeonol on no-reflow and regional blood perfusion during myocardial I/R in rat models. We hypothesize that paeonol exerts cardioprotective effects by attenuating I/R-associated no-reflow injuries and therefore may be considered as a potential preventive treatment for cardiac diseases or post-coronary revascularization when no-reflow occurs.

## Materials and methods

### Experimental animals and reagents

Healthy male Wistar rats (180–220 g, average age of 8 week) were procured from Beijing Si Beifu Laboratory Animal Technology Co., Ltd. All animal experiments were performed in accordance with the standards established by the Institutional Animal Care and Use Committee of Institute of Basic Medical Sciences of Xiyuan Hospital, and were approved by the Beijing Local Ethics Review Committee, Beijing University Laboratory Animal Center (Animal Permit #: SCXK Beijing 2011-0004). The rats were randomly divided into four groups: (1) sham group, thoracotomy without left anterior descending coronary artery (LAD) occlusion or paeonol pretreatment; (2) I/R group, LAD occlusion (ischemia) for 4 h followed by reperfusion for 8 h; (3) Paeonol (100 mg/kg) + I/R group, oral administration of 100 mg/kg paeonol (1 mL/kg) for 7 days using a intragastric tube prior to I/R procedure; (4) Paeonol (200 mg/kg) + I/R group, oral administration of 200 mg/kg paeonol (1 mL/kg) for 7 days using a intragastric tube prior to I/R procedure. In addition, rats in the sham and I/R groups received a dosage of dimethyl sulfoxide (DMSO, Beijing Solarbio Science & Technology Co., Ltd., China) equal to that with which the paeonol was dissolved in for the other two groups. DMSO was also administered intragastrically for 7 consecutive days. A minimum of eight rats were assigned to each group (Zhang et al., 2014). An ischemia group without reperfusion is not included since our present study mainly focuses on the effect of paeonol on the cardiac injuries after reperfusion, which is closely related to the real-world situation of no-reflow after coronary revascularization. However, future studies may include a group subjected only to 4 h of ischemia to differentiate, in terms of damage to the cardiac function, which was due to the ischemia and which was due to the no-reflow.

Previous literature reported a dose-dependent (30, 50, 100 mg/kg) effect of paeonol in reducing inflammation (Chou, 2003). In our myocardial I/R models, 100 mg/kg paeonol was used as low-dose and 200 mg/kg as high-dose to ensure a range of dosage effectiveness. In addition, to our best knowledge, the duration of drug action for paeonol has not yet been fully investigated. Taken into account of this, we designed a 7-day administration period of pretreatment to ensure a sufficient time for the onset of paeonol. Future studies will focus on the drug action as well as the dosage effectiveness of paeonol.

Paeonol (purity > 95%) was obtained from Ningbo Dekang Biologic Product Co., Ltd. (Ningbo, China). Nitro-blue tetrazolium chloride (NBT) and chloral hydrate were purchased from Sinopharm Chemical Reagent Co., Ltd. (Beijing, China). Thioflavin S was purchased from Sigma (St. Louis, MO, USA). Lactate dehydrogenase (LDH), creatine kinase (CK), cardiac troponin T (cTnT), and C-reactive protein (CRP) assay kits were purchased from BioSino Bio-technology and Science Inc. (Beijing, China). All of the chemicals were dissolved in physiological saline, except for paeonol, which was dissolved in DMSO.

### Establishment of myocardial I/R rat model

The myocardial I/R model was established previously by Michael et al. (1995). Briefly, rats were anesthetized via an intraperitoneal (IP) injection of 3.5% chloral hydrate (1 mL/kg) and placed in a supine position with all paws secured on the operating table. The left thorax was opened to expose the heart. The LAD coronary artery was ligated and the chest was closed by a hemostat for 4 consecutive hours until the reperfusion took place (Zhang et al., 2014). Although in rodent heart, 30–60 min ischemia is normally sufficient to induce myocardial infarction, ischemia for 4 h in our setting would lead to a more severe risk region in order to fully evaluate the therapeutic efficacy of paeonol during no-reflow, as described previously (Zhang et al., 2014). Compared to models with relatively shorter ischemia periods, no-reflow area and perfusion area were more clearly identified in our unique 4 h ischemic model, and the heart remained viable after this prolonged ischemia caused by LAD ligation since other coronary arteries were still able to partially support the blood flow to the left ventricle (Zhang et al., 2014). Following the removal of LAD ligation during another chest opening for reperfusion, the chest was re-sutured and the analysis was performed 8 h later from the start of reperfusion. In the sham group, the rat underwent the same chest surgery in the absence of LAD ligation. The body temperature of the rats was maintained at all time. The chest opening time during LAD occlusion and ligation removal was kept at minimum by experienced surgeons. Successful induction of acute myocardial I/R model was confirmed by visual inspection of color alteration in the LV and ST segment-characterized electrocardiogram.

### Cardiac function by echocardiography

Following 8 h of reperfusion, rats were adequately sedated by 3% isoflurane (inhaled). Ultrasound gel was placed on the precordial region of the shaved chest wall, and ultrasound bio-microscopy (Vevo 770, Visual-Sonics Inc., Toronto, ON, Canada) with a 17.5-MHz transducer was used to visualize the left ventricle. The LV wall thickness, chamber diameter, and pressure were measured from the two-dimensional directed images of the LV (M-mode; short axis below papillary muscle), as previously described (Samuel et al., 2008). LV function was assessed by measuring LV end-systolic and end-diastolic volumes, ejection fraction (EF), stroke volume (SV), and fractional shortening (FS). All measurements were recorded from at least three consecutive cardiac cycles and the mean values were reported.

### Myocardial contrast echocardiography (MCE) analysis

Real-time MCE was used to capture MCE sequences during cardiac cycles and quantitatively analyze the extent of myocardial blood flow. Regions of interest (ROIs) were positioned at the anterior wall of LV to measure the non-perfused areas in our models. Constant infusion of microbubble contrast agent (0.8 ml of 11.8 mg/ml per mouse) was performed via tail vein injection. The total imaging acquisition lasted for ~12.5 s. The signal intensity increased immediately following the injection of contrast agents. The displayed pattern of the curve included an ascending slope followed by a plateau, indicating an increase followed by stabilization of the detectable intensity from contrast agents. The time-dependent perfusion in the heart can be described with a mathematical model, which can be solved using nonlinear regression analysis. The exponential curve of time versus intensity was then graphed based on previous studies (Raher et al., 2007; Sun et al., 2011).

Signal variation is modeled as *y* = A (1 − *e*^βt^), where *y* is the contrast agent replenishment data at any given time. A (plateau intensity) and β (rate constant of signal intensity rise) represent microvascular cross-sectional area and blood velocity, respectively. These values are automatically generated by MCE software. On the other hand, the calculated product A·β provides an estimation of myocardial blood flow (Wei et al., 1998; Raher et al., 2007).

### Measurement of serum parameters

After the completion of cardiac function assessment and MCE analysis, thioflavin S (6%, 1 mL/kg) was injected into the femoral vein of all rats for the subsequent examination of the infarct area. Blood was immediately collected from the abdominal aorta and centrifuged at 1050 × *g* for 2 × 10 min. The serum was analyzed using assay kits to detect the activities of LDH, CK, cTnT, and CRP. All procedures were performed according to the manufacturer's protocol (Goldberg et al., 2006; Xia et al., 2014).

### Myocardial staining for no-reflow and infarct area evaluation

Rats were sacrificed immediately by removing the whole heart. The heart was transected into five even slices parallel to the atrioventricular groove. The slices were photographed under ultraviolet light (UV, 365 nm) to identify the region of no-reflow. By thioflavin S staining, areas that were perfused with blood were fluorescent, whereas areas that were not perfused appeared dark (Shao et al., 2013). The slices were then incubated in 0.2% NBT for 3 min in room temperature, immersed in formalin, and photographed again. Infarct area was unstained by NBT and the non-infarct area was stained dark brown (Kloner et al., 1974). The no-reflow and infarct areas were measured by a multimedia color pathological image analytical system (MPIAS-500, Beijing Kong Hai Science and Technology Development Co., Ltd., China). Data of no-reflow area were determined as the percentage of the no-reflow area to the ventricle size (AN/V) or the no-reflow area to the whole heart section (AN/WH). Data of infarct area were determined as the percentage of the infarct area to the ventricle size (AI/V) or the infarct area to the whole heart section (AI/WH; Shao et al., 2013).

### Statistical analysis

Data were analyzed using SPSS 17.0 software (SPSS Inc., Chicago, IL, USA) and the values are expressed as mean ± standard error (SE). A normality test was performed on all presented data and the differences between each group were assessed by nonparametric Mann-Whitney test. The results were considered significant at *p* < 0.05.

## Results

### Effects of paeonol on myocardial infarct area

**Figure 1:**
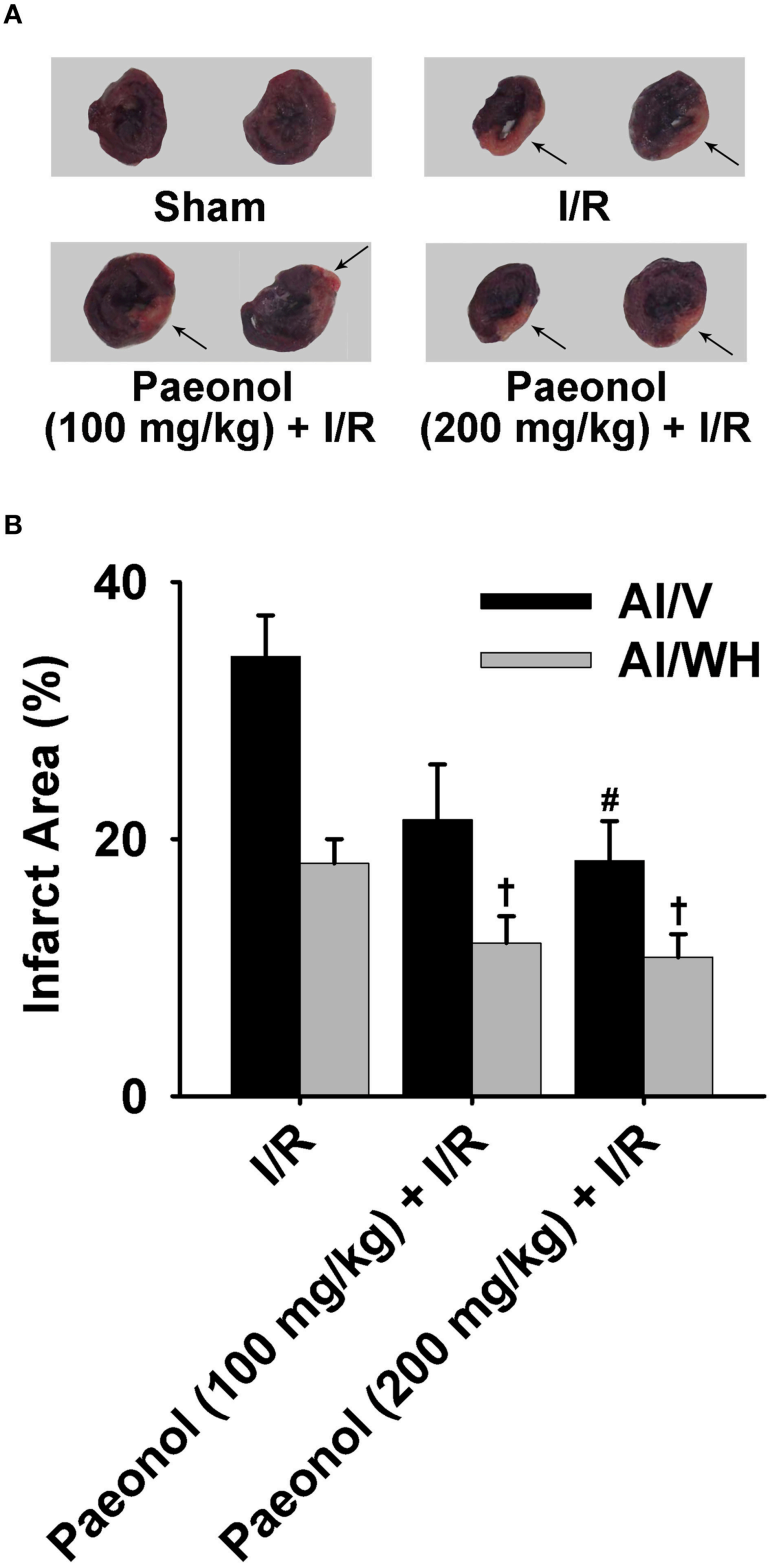
**Effects of paeonol on myocardial infarct area in ischemia/reperfusion (I/R) rats. (A)** Representative images of the infarct area for all four groups [Sham, I/R, paeonol (100 mg/kg) + I/R, paeonol (200 mg/kg) + I/R], with the arrows showing nitro-blue tetrazolium chloride (NBT)-negative infarct areas. **(B)** The percentage of the infarct area to the ventricle size (AI/V) and to the whole heart section (AI/WH) in the three groups. I/R group (*n* = 9), paeonol (100 mg/kg) + I/R group (*n* = 8), paeonol (200 mg/kg) + I/R group (*n* = 8). The data are expressed as mean ± SE. ^#^*p* < 0.01, compared with I/R group of the ventricle size. ^†^*p* < 0.05, compared with I/R group of the whole heart.

Initial *in vivo* experiment of paeonol was performed in rat models of myocardial I/R induced by LAD ligation. Different doses of paeonol (100 and 200 mg/kg) were used during the pretreatment of two experimental groups. Myocardial injury was evident by the quantitative measurement of acute myocardial infarct sizes. The 200 mg/kg paeonol + I/R group [AI/V (%): 18.3 ± 3.1, *p* < 0.01; AI/WH (%): 10.8 ± 1.8, *p* < 0.05] and 100 mg/kg paeonol + I/R group [AI/WH (%): 11.9 ± 2.1, *p* < 0.05] exhibited significant reductions in the infarct area compared with the I/R group [AI/V (%): 34.2 ± 3.2; AI/WH (%): 18.1 ± 1.9] (Figure 1, Datasheet 2). However, the comparison between the 100 mg/kg paeonol + I/R group and the 200 mg/kg paeonol + I/R group showed no significant difference in the infarct area reduction (AI/V, *p* > 0.05; AI/WH, *p* > 0.05).

### Effects of paeonol on no-reflow area

**Figure 2:**
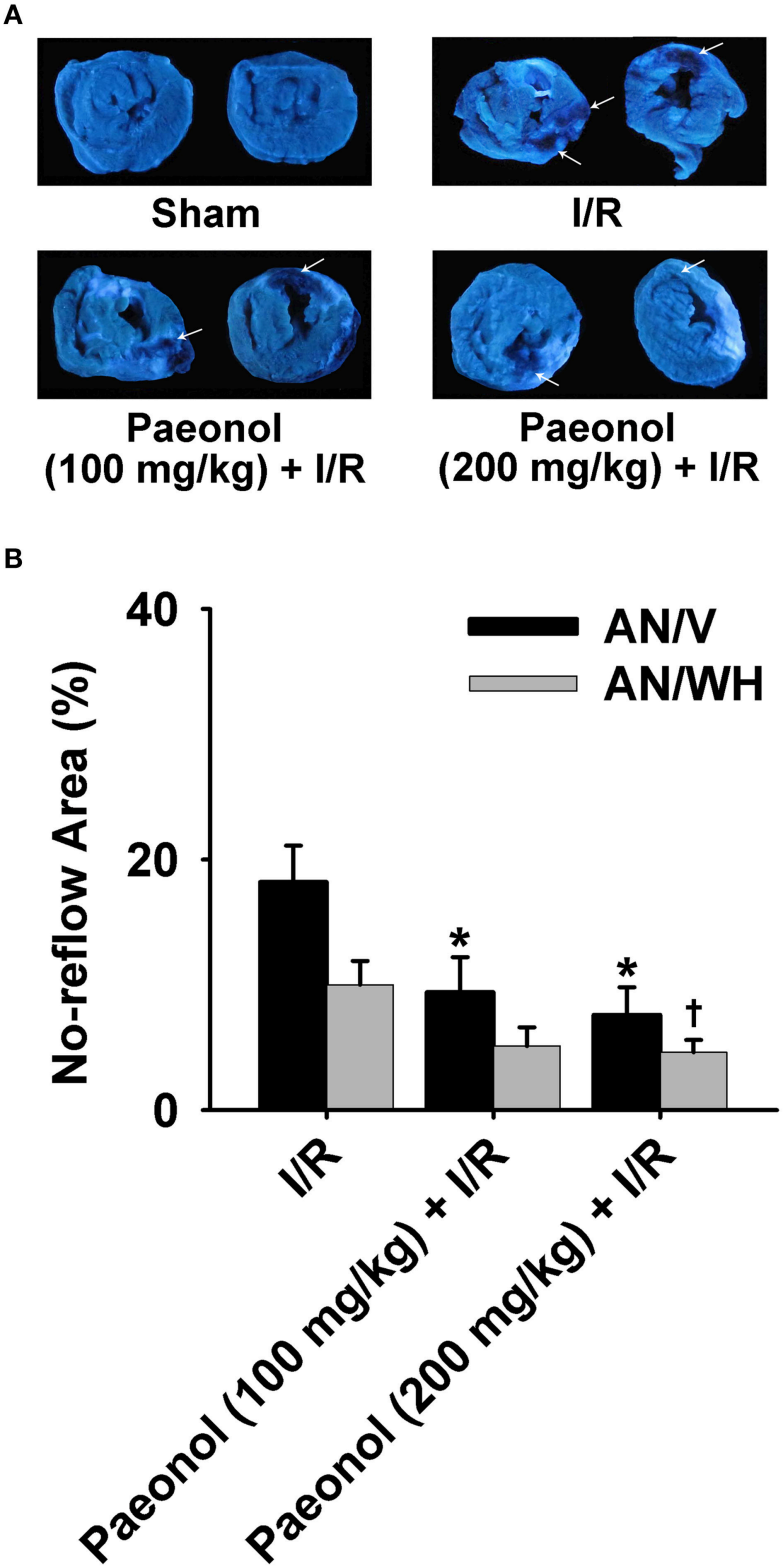
**Effects of paeonol on no-reflow area in myocardial ischemia/reperfusion (I/R) rats. (A)** Representative images of the no-reflow area for all four groups [Sham, I/R, paeonol (100 mg/kg) + I/R, paeonol (200 mg/kg) + I/R], with the arrows showing no-reflow areas. **(B)** The percentage of the no-reflow area to the ventricle size (AN/V) and to the whole heart section (AN/WH) in the three groups. I/R group (*n* = 9), paeonol (100 mg/kg) + I/R group (*n* = 8), paeonol (200 mg/kg) + I/R group (*n* = 8). The data are expressed as mean ±SE. ^*^p < 0.05, compared with I/R group of the ventricle size. ^†^*p* < 0.05, compared with I/R group of the whole heart.

The 200 mg/kg paeonol + I/R group [AN/V (%): 7.6 ± 2.2, *p* < 0.01] and 100 mg/kg paeonol + I/R group [AN/V (%): 9.4 ± 2.8, *p* < 0.05] both showed lesser extents of no-reflow area in the ventricles compared with the I/R group [AN/V (%): 18.2 ± 2.9]. In particular, the 200 mg/kg paeonol + I/R group experienced markedly alleviated no-reflow in the whole heart [AN/WH (%): 4.6 ± 1.0, *p* < 0.05] compared with the I/R group [AN/WH (%): 10.0 ± 1.9] (Figure 2, Datasheet 4). Furthermore, no significant difference in no-reflow area was found between the two dosages of paeonol (AN/V, *p* > 0.05; AN/WH, *p* > 0.05).

### Effects of paeonol on regional myocardial blood flow

**Figure 3:**
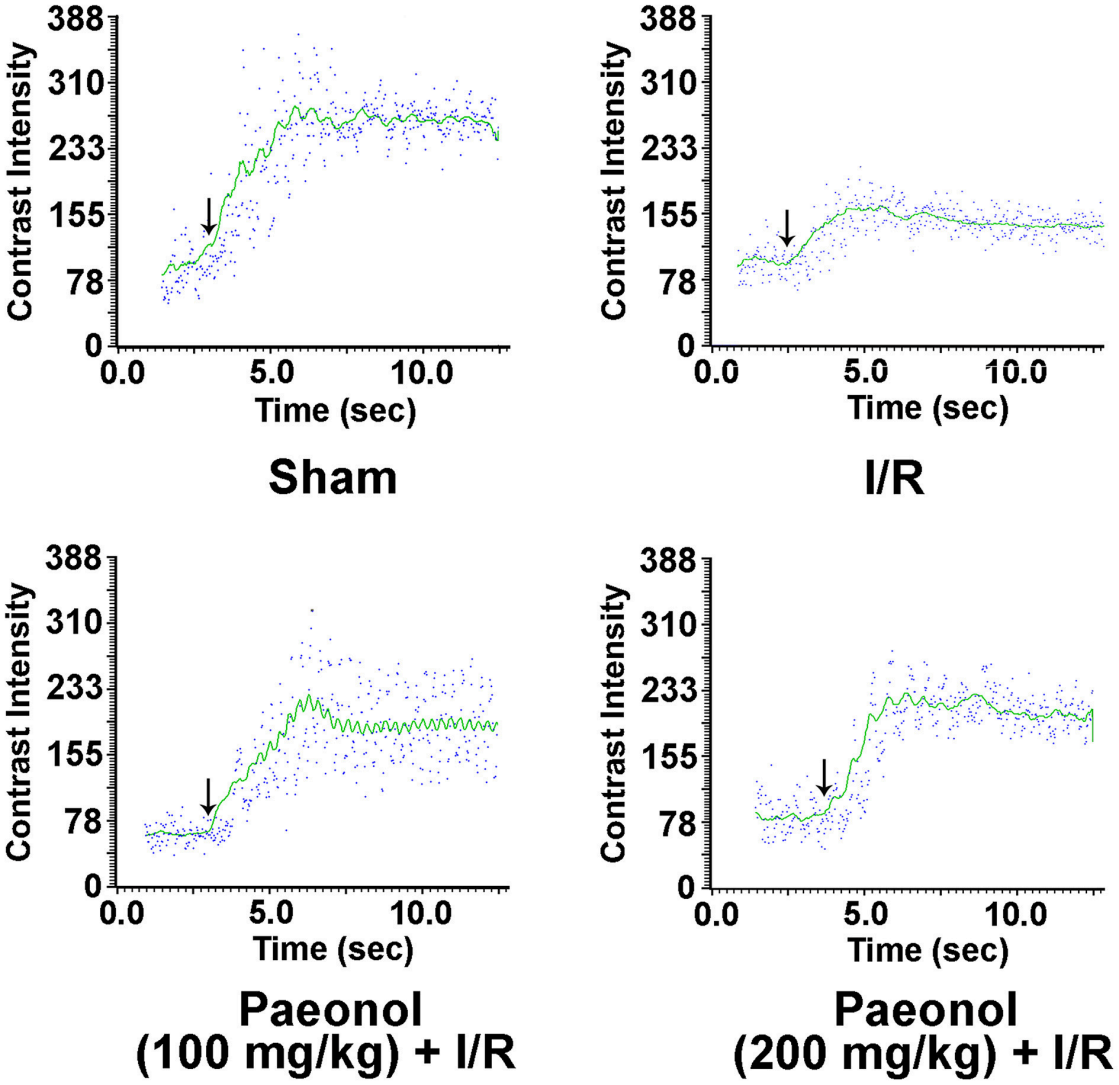
**Effect of paeonol on regional myocardial blood flow**. Representative images of regional myocardial blood flow of the four groups. Sham group (*n* = 9), I/R group (*n* = 9), paeonol (100 mg/kg) + I/R group (*n* = 8), paeonol (200 mg/kg) + I/R group (*n* = 8). The arrows indicate the time point for the contrast agent injection.

The quantitation of paeonol's effect on the regional myocardial blood flow was assessed by MCE (Figure 3 and Table 1). The results showed that β (0.28 ± 0.03 /s, *p* < 0.05), A (102.21 ± 11.69 dβ, *p* < 0.01) and A·β (26.76 ± 2.39 dβ/s, *p* < 0.001) were significantly decreased in the I/R group compared with the sham group (0.42 ± 0.05 /s, 193.33 ± 13.34 dβ, 86.32 ± 18.65 dβ/s), respectively. In the paeonol-pretreated groups, β (200 mg/kg paeonol group: 0.43 ± 0.05 /s, *p* < 0.05; 100 mg/kg paeonol group: 0.55 ± 0.11 /s, *p* < 0.01), A (200 mg/kg paeonol group: 173.79 ± 15.99 dβ, *p* < 0.01; 100 mg/kg paeonol group: 183.83 ± 17.01 dβ, *p* < 0.01) and A·β (200 mg/kg paeonol group: 70.01 ± 7.06 dβ/s, *p* < 0.001; 100 mg/kg paeonol group: 87.32 ± 14.82 dβ/s, *p* < 0.001), were significantly increased as compared to that in the I/R group (Table 1, Datasheet 3). However, no significant dose-dependent difference in MCE results was observed between two doses of paeonol (100 mg/kg vs. 200 mg/kg; β, *p* > 0.05; A, *p* > 0.05; A·β, *p* > 0.05).

**Table 1:** **Quantitative MCE analysis of regional myocardial blood flow**.

	**A (dB)**	**β (s^−1^)**	**A·β (dB/s)**
**Groups**			
Sham (*n* = 9)	193.33 ± 13.34	0.42 ± 0.05	86.32 ± 18.65
I/R (*n* = 9)	102.21 ± 11.69^Δ^	0.28 ± 0.03^‡^	26.76 ± 2.39^§^
Paeonol (100 mg/kg) + I/R (*n* = 8)	183.83 ± 17.01^#^	0.55 ± 0.11^#^	87.32 ± 14.82^†^
Paeonol (200 mg/kg) + I/R (*n* = 8)	173.79 ± 15.99^#^	0.43 ± 0.05^*^	70.01 ± 7.06^†^

A, plateau; β rise rate of the signal intensity; A·β ± regional myocardial perfusion.

Values are mean ± SE.

* p < 0.05,

# p < 0.01,

† *p* < 0.001 vs. I/R group.

‡ *p* < 0.05,

Δ p < 0.01,

§ *p < 0.001 vs. sham group*.

### Effects of paeonol on cardiac function in myocardial I/R rats

**Figure 4:**
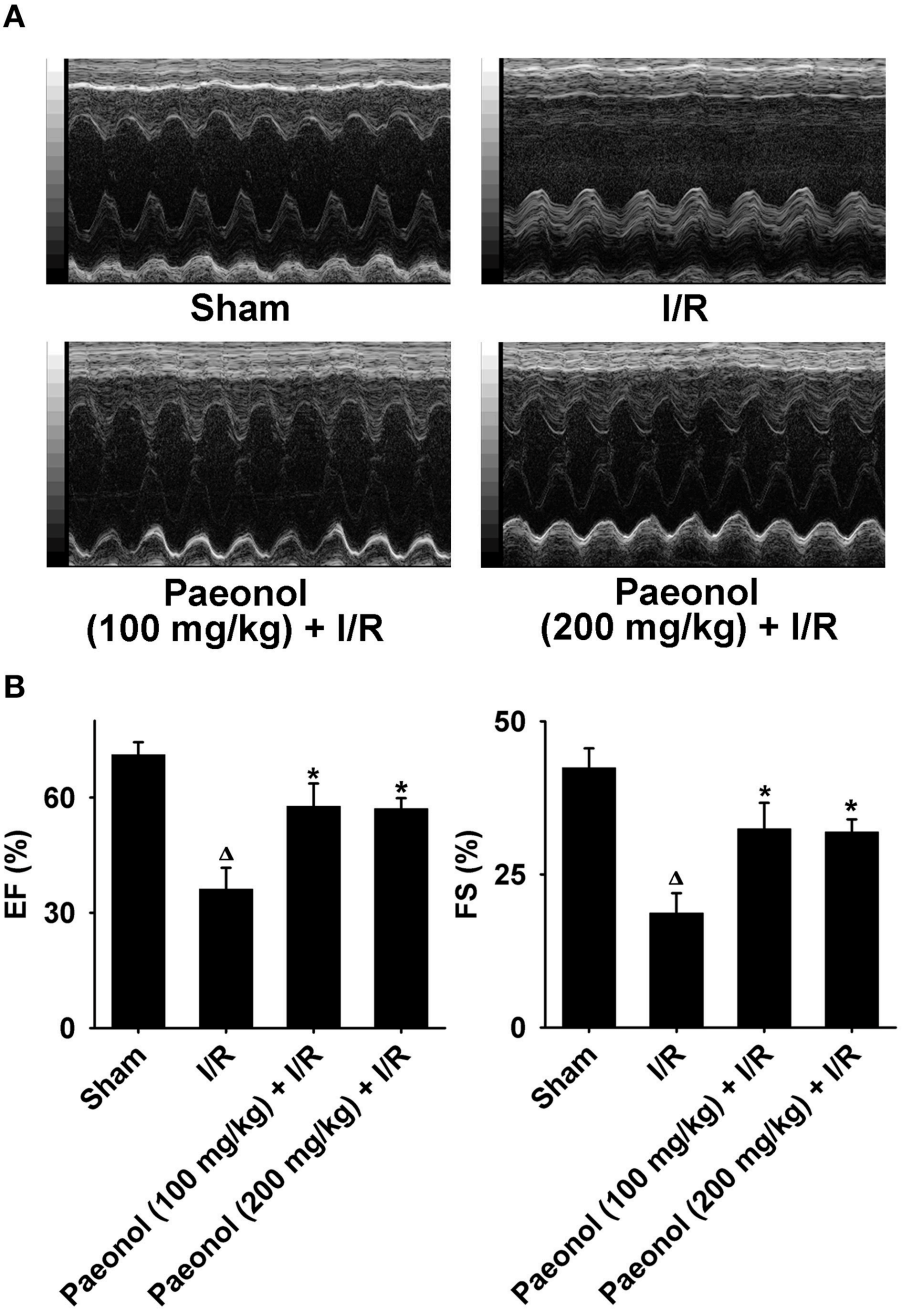
**Effect of paeonol on cardiac function evaluated by echocardiography. (A)** Representative echocardiographic images of the four groups after the surgical procedure. Each image shown is 1-s duration of the entire echocardiograph. **(B)** Percentage of ejection fraction (EF) and fractional shortening (FS) affected by paeonol. Sham group (*n* = 9), I/R group (*n* = 9), paeonol (100 mg/kg) + I/R group (*n* = 8), paeonol (200 mg/kg) + I/R group (*n* = 8). ^*^p < 0.05 compared with I/R group. ^Δ^*p* < 0.001 compared with sham group.

We also examined whether paeonol alters cardiac functional parameters in myocardial I/R rats using echocardiographic recording (Figure 4). The LV anterior wall systolic and diastolic thicknesses (LVAW d/s) were significantly improved in the 200 mg/kg paeonol + I/R group (*n* = 8; *p* < 0.05) and the 100 mg/kg paeonol + I/R group (LVAW d; *n* = 8; *p* < 0.01) compared with that in the I/R group. However, no marked differences in the LV posterior wall diastolic and systolic thickness (LVPW d/s) were observed. The LV internal diameter in systole (LVID s) was reduced only in the 200 mg/kg paeonol-pretreated + I/R group (*n* = 8; *p* < 0.01), and LVID in diastole (LVID d) in both paeonol-pretreated groups showed no significant difference when compared to I/R group. The LV volume in systole (LVV s) was significantly decreased (*n* = 8; *p* < 0.05) in both 200 mg/kg paeonol + I/R group and 100 mg/kg paeonol + I/R group, whereas LVV in diastole (LVV d) expressed no marked difference (Table 2). Along with elevated EF and FS in both paeonol-pretreated groups (*n* = 8; *p* < 0.05; Table 2), these results showed improved cardiac function with paeonol treatment. Interestingly, greater SV was observed only in the 200 mg/kg paeonol + I/R group (*n* = 8; *p* < 0.05), yet no significant difference was seen in the 100 mg/kg paeonol + I/R group (Table 2, Datasheet 1).

**Table 2:** **Echocardiographic analysis of cardiac function in four groups**.

	**Sham**	**I/R**	**Paeonol (100 mg/kg) + I/R**	**Paeonol (200 mg/kg) + I/R**
n	9	9	8	8
LVAW d (mm)	1.89 ± 0.07	1.49 ± 0.08^‡^	1.86 ± 0.12^#^	1.89 ± 0.10^*^
LVAW s (mm)	2.75 ± 0.10	1.63 ± 0.11^Δ^	1.97 ± 0.10^*^	2.11 ± 0.08^#^
LVPW d (mm)	1.90 ± 0.08	1.87 ± 0.09	1.86 ± 0.11	2.02 ± 0.07
LVPW s (mm)	2.67 ± 0.11	2.21 ± 0.23	2.58 ± 0.20	2.84 ± 0.28
LVID d (mm)	6.70 ± 0.15	7.64 ± 0.29^†^	7.34 ± 0.36	7.03 ± 0.14
LVID s (mm)	3.91 ± 0.27	6.30 ± 0.41^Δ^	5.25 ± 0.50	4.84 ± 0.19^#^
LVV d (μl)	236.10 ± 10.77	316.18 ± 26.06^†^	253.31 ± 23.84	257.92 ± 13.69
LVV s (μl)	70.32 ± 9.71	208.88 ± 30.55^‡^	116.07 ± 26.36^*^	110.55 ± 10.68^#^
EF (%)	71.13 ± 3.25	36.17 ± 5.53^Δ^	57.73 ± 5.91^*^	57.09 ± 2.73^*^
FS (%)	42.41 ± 3.17	18.68 ± 3.25^Δ^	32.44 ± 4.24^*^	31.92 ± 2.08^*^
SV (μl)	165.78 ± 4.76	107.30 ± 12.89^Δ^	137.24 ± 5.76	147.37 ± 7.71^*^

LVAW d/s, left ventricle anterior wall diastolic and systolic thicknesses; LVPW d/s, left ventricle posterior wall diastolic and systolic thickness; LVID d/s, left ventricle internal diameter in diastole and systole; LVV d/s, left ventricle volume in diastole and systole; EF, ejection fraction; FS, fractional shortening; SV, stroke volume.

Values are mean ± SE.

* p < 0.05,

# p < 0.01 vs. I/R group.

† p < 0.05,

‡ p < 0.01,

Δ *p < 0.001 vs. sham group*.

### Effects of paeonol on serum LDH, CK, cTnT, and CRP levels in myocardial I/R rats

**Figure 5:**
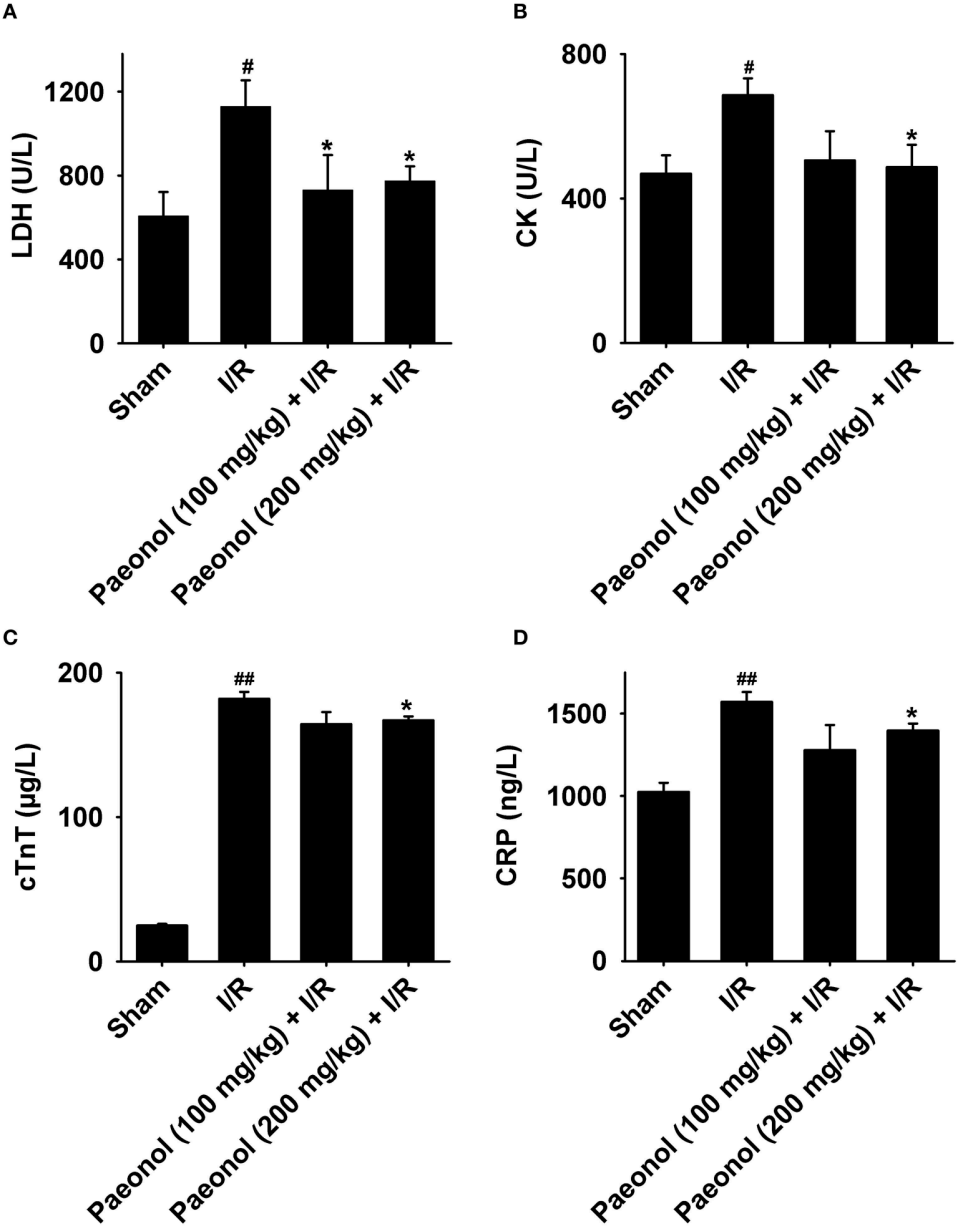
**Effects of paeonol on the levels of serum biomarkers, lactate dehydrogenase (LDH; A), creatine kinase (CK; B), cardiac troponin T (cTnT; C), and C-reactive protein (CRP; D), in the four groups**. Sham group (*n* = 9), I/R group (*n* = 9), paeonol (100 mg/kg) + I/R group (*n* = 8), paeonol (200 mg/kg) + I/R group (*n* = 8). The data are expressed as mean ± SE. ^*^p < 0.05, compared with I/R group; ^#^*p* < 0.05, ^##^*p* < 0.001, compared with sham group.

LDH, CK, cTnT, and CRP concentrations were all elevated in the I/R group [LDH (U/L): 1128.67 ± 124.80, *p* < 0.05; CK (U/L): 686.28 ± 46.25, *p* < 0.05; cTnT (μg/L): 181.94 ± 4.72, *p* < 0.001; CRP (ng/L): 1570.96 ± 60.31, *p* < 0.001] compared with the sham group [LDH (U/L): 607.11 ± 114.35; CK (U/L): 468.56 ± 50.93; cTnT (μg/L): 24.89 ± 1.16; CRP (ng/L): 1024.18 ± 55.45]. In the paeonol-pretreated groups, a marked reduction in LDH concentration was observed in 100 mg/kg paeonol + I/R group [LDH (U/L): 730.28 ± 167.25]; yet 200 mg/kg paeonol + I/R group [LDH (U/L): 774.00 ± 69.97; CK (U/L): 486.99 ± 61.83; cTnT (μg/L): 167.03 ± 2.63; CRP (ng/L): 1396.79 ± 42.88] significantly declined all four biomarker measurements compared with the I/R group (*n* = 9; *p* < 0.05, Figures 5A–D, Datasheet 5). Moreover, the comparison between the two paeonol-pretreated groups showed no significant difference in LDH, CK, cTnT, and CRP levels (*p* > 0.05).

## Discussion

No-reflow phenomenon emerges when blood fails to reperfuse an ischemic area after coronary revascularization (Shao et al., 2013). When no-reflow occurs, pharmacological treatments including intracoronary verapamil and adenosine, as well as mechanical therapies (e.g., aspiration thrombectomy) can be used to partially relieve microvasculature obstruction and improve blood flow in the heart. However, the development of a more efficient treatment, especially the prevention of no-reflow, still remains extremely challenging due to the complexity of this phenomenon (Ramjane et al., 2008; Berg and Buhari, 2012). As one of the active compounds in *P. suffruticosa*, paeonol has been shown to exert beneficial effects, such as vascular dilation and arrhythmia improvements (Li Y.J. et al., 2010; Li et al., 2012). Our findings of the effect of paeonol on regional blood perfusion in the present study are consistent with the aforementioned reports. In particular, our results further indicated that pretreatment with paeonol reduced no-reflow area in conjunction with marked decreased release of serum CK and LDH, as well as a slight decline of cTnT and CRP. Our study is the first to show that paeonol pretreatment has pharmacologically protective effects on the no-reflow phenomenon. This result, consistent with our hypothesis, provides evidence that using paeonol may be a preventive strategy for no-reflow injuries in cardiac diseases or post-coronary revascularization, warranting pre-clinical testing in other models.

Myocardial infarction and no-reflow are the major manifestations of reperfusion injury (Kloner et al., 2012). Our data showed that paeonol significantly minimized the no-reflow area. Consequently, the same trend was observed in the infarct area, indicating that the extent of no-reflow was likely correlated with the extent of infarction, consistent with literature (Coggins et al., 2001). Furthermore, we observed a reduction in the release of LDH, CK, cTnT, and CRP, all of which are indicators of potential myocardial injuries in 200 mg/kg paeonol-pretreated rat group, although no significant dose-dependent difference is observed between the two doses of paeonol. LDH and CK are important markers of cellular necrosis (Xia et al., 2014). Elevated CK and cTnT levels, which are often associated with systemic inflammation, have been detected in angina patients undergoing PCI (Goldberg et al., 2006). Thus, our results suggested that the paeonol treatment may alleviate myocardial injury in rat models at molecular levels.

Real-time MCE is a promising echocardiography technology that is clinically applied for the noninvasive evaluation of myocardial capillary perfusion in patients (Agati et al., 2004). Given that myocardial infarctions are closely related to the insufficient blood supply (Wagner et al., 2002; Thygesen et al., 2007), we examined regional myocardial blood flow by MCE in myocardial I/R rats. We found that paeonol significantly increased the plateau (A), rise rate of the signal intensity (β), and regional myocardial perfusion (A·β) in both paeonol-pretreated groups. These observations showed that paeonol pretreatment reduced myocardial ischemic damage most likely via a potential modulation of microvasculature. In the long run, paeonol may enhance the blood supply of impaired microvessels, thereby impeding myocardial I/R-associated no-reflow. Based on our results, LVAW, EF, FS, and SV were dramatically elevated, whereas LVID s and LVV s were markedly decreased compared with the groups with no paeonol pretreatment, benefitting cardiac performance. However, some cardiac functions including LVID d and LVV d showed no significant difference in the paeonol-pretreated groups compared to I/R groups, which is likely due to the use of non-optimal dosage of paeonol and/or insufficient duration of administration in the present study. Notably, no marked difference in certain measured parameters (including infarct area, no-reflow area, regional myocardial blood flow and cardiac function) was found between the two doses of paeonol, indicating the importance of evaluating and determining the dose effectiveness, administration duration, and onset of paeonol.

The upregulation of Bcl-2 family proteins signals cell death under I/R stress, but the effect is diminished upon paeonol treatment (Li et al., 2012). Indeed, the most studied pharmacological intervention for no-reflow, verapamil, is a calcium channel blocker that improves coronary blood flow (Berg and Buhari, 2012). Previous studies have shown that paeonol induces vasodilatation of the rat mesenteric arteries by regulating intracellular Ca^2+^ concentration and improves Ca^2+^ATPase activity in the ischemic tissue (Zhang et al., 1997, 2013; Yuan and Jing, 2011). It is possible that paeonol suppresses both Ca^2+^ overload and hyper-contracture, thus eliciting beneficial effects on cardiac function. However, the exact molecular mechanisms underlying the protective effect of paeonol in no-reflow have not been fully elucidated. Particularly, Li et al. observed an enhanced antioxidant defense in the paeonol-treated rat group, suggesting Nrf2 activation may be involved in the upregulation of paeonol's cardioprotective signaling cascades (Li et al., 2012). Since reactive oxygen species have been shown to play a critical role in almost all biological systems and pathological conditions, including I/R injuries, further research could be focused on the redox mechanism initiated by paeonol (Zuo et al., 2011a,b, 2014, 2015a,b).

We explored the preventive effect of paeonol on no-reflow injuries, which commonly occurred after coronary revascularization. Therefore, the administration of paeonol before any potential cardiac attack or at a daily basis may still be a feasible real-world prevention. Limitations of this study may include the fact that the exact molecular mechanisms of paeonol's cardioprotective effects associated with multiple cardiac variables are still not fully understood at the current stage of the study. Our experiment has not yet fully identified the optimal dosages for paeonol's protection to the heart. We did not find any significance of infarct area, no-reflow area, and regional myocardial blood flow between the two doses of paeonol, suggesting the necessity for further experiments to determine the dose effectiveness and administration strategies for paeonol. Moreover, since the paeonol treatment does not have any standard pharmacological treatments in our analyzed variables, future efforts may be focused on the optimal dosage effectiveness as well as the duration of drug action of paeonol.

## Conclusion

This study has shown the functional cardioprotective properties of paeonol *in vivo* rat model. Paeonol minimized the sizes of myocardial infarct area and no-reflow area, and improved regional myocardial blood flow and cardiac function. Our results demonstrate that paeonol may be an alternative drug for clinical application in the prevention of myocardial I/R-associated no-reflow and other related cardiac diseases.

## Author contributions

Conception and design of research: WW. Performed experiments: LeZ, JZ. Analyzed data: LM, YZ, JZ, CC, LiZ. Interpreted results of experiments: LM, JZ, CC, LiZ. Prepared figures: LM, CC, LiZ. Drafted manuscript: LM, CC, LiZ. Edited and revised manuscript: LiZ. Approved final version of manuscript: LM, CC, WW, LeZ, YZ, JZ, LiZ.

## Funding

This study was supported by China Postdoctoral Science Foundation Fund (2012T50202), Beijing Joint Project Special Funds, as well as OSUCOM-HRS fund 013000 (USA).

### Conflict of interest statement

The authors declare that the research was conducted in the absence of any commercial or financial relationships that could be construed as a potential conflict of interest.
