# Supplementary material for: Paeonol Protects Rat Heart by Improving Regional Blood Perfusion during No-Reflow
Source: Front Physiol. 2016 Jul 21;7:298. doi: 10.3389/fphys.2016.00298 (PMC4954854; doi:10.3389/fphys.2016.00298)

**Infarct size statistical analysis (Normality test and Nonparametric test)**


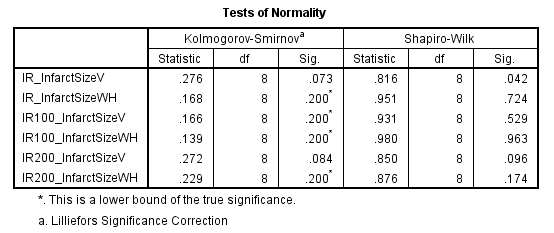


NOTE: Shapiro-Wilk Test is more appropriate for small sample sizes (< 50 samples).

Since the data for infract size in ventricle in the I/R group is not normally distributed (<0.05; red box), a nonparametric test is performed.

**Nonparametric tests (two-independent samples, Mann-Whitney test)**

Treatment groups: 1) I/R group, 2) I/R + paeonol 100 mg/kg, 3) I/R + paeonol 200 mg/kg


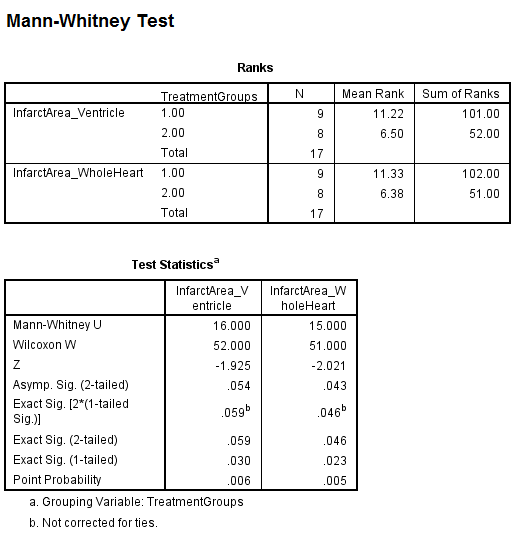


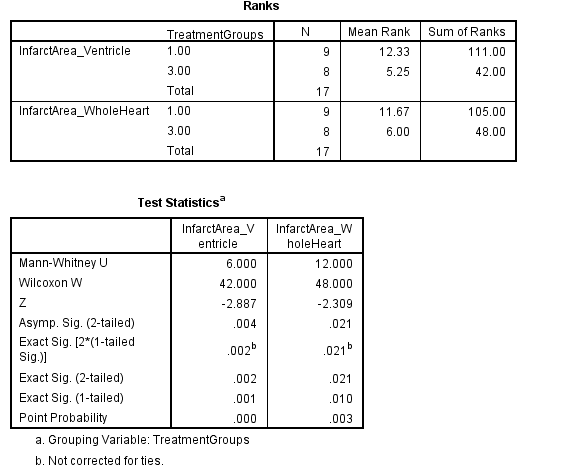


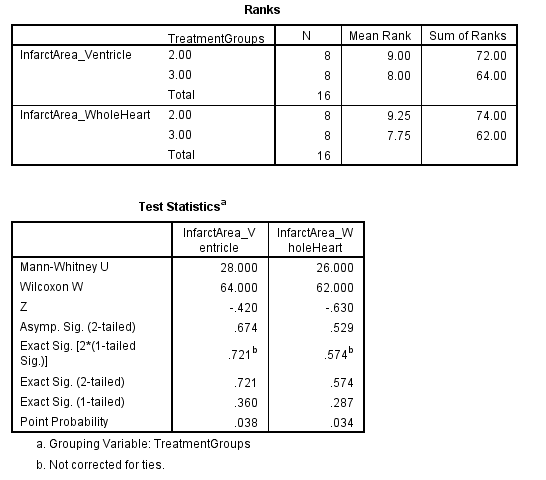


Mean ± SE and SD


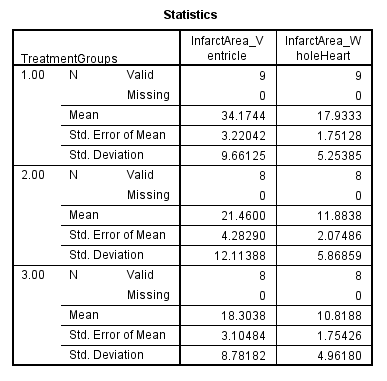

Supplement: Supplementary file 2 [file DataSheet2.DOCX]
