# Supplementary material for: Paeonol Protects Rat Heart by Improving Regional Blood Perfusion during No-Reflow
Source: Front Physiol. 2016 Jul 21;7:298. doi: 10.3389/fphys.2016.00298 (PMC4954854; doi:10.3389/fphys.2016.00298)

**No-reflow statistical analysis (Normality test and Nonparametric test)**


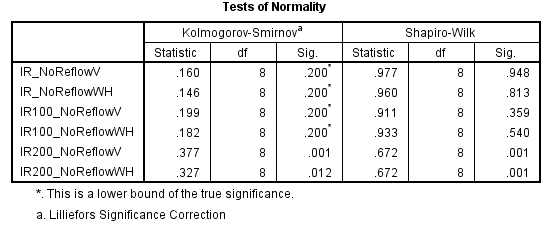


The data for no reflow in ventricle and whole heart in the I/R paeonol + 200 mg/kg group is not normally distributed (<0.05; red box). Nonparametric tests are performed.

**Nonparametric tests (two-independent samples, Mann-Whitney test)**

Treatment groups: 1) I/R group, 2) I/R + paeonol 100 mg/kg, 3) I/R + paeonol 200 mg/kg


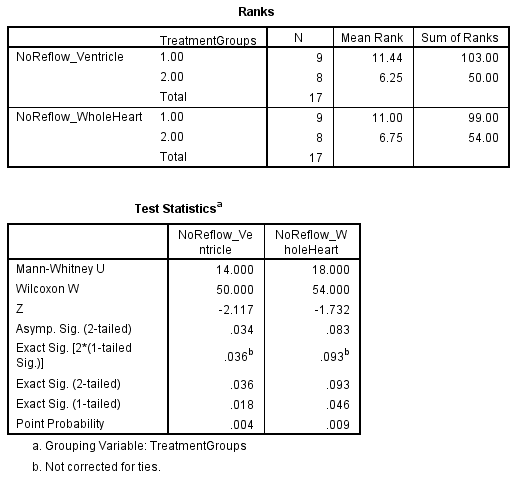


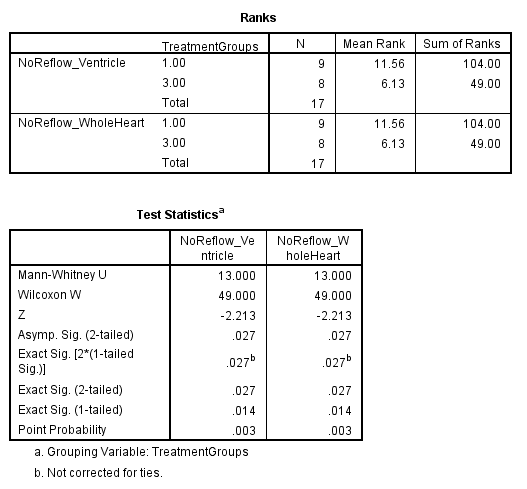


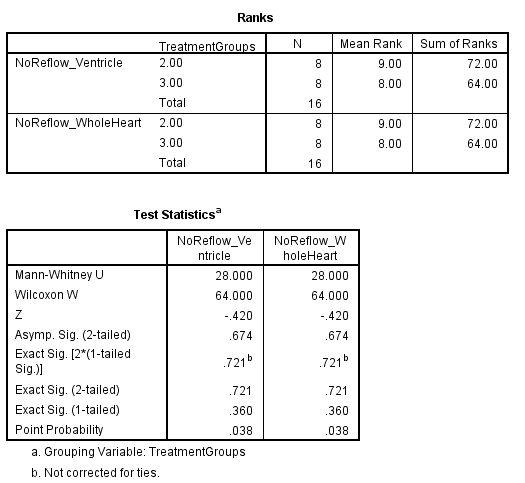


Mean ± SE and SD


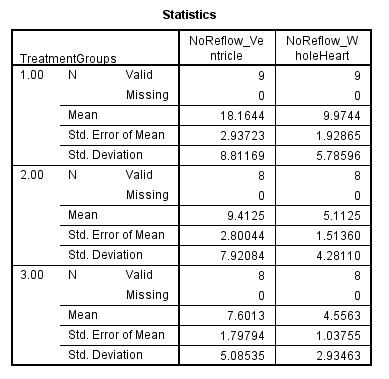

Supplement: Supplementary file 4 [file DataSheet4.DOCX]
