# Supplementary material for: Paeonol Protects Rat Heart by Improving Regional Blood Perfusion during No-Reflow
Source: Front Physiol. 2016 Jul 21;7:298. doi: 10.3389/fphys.2016.00298 (PMC4954854; doi:10.3389/fphys.2016.00298)

**Serum parameter statistical analysis (Normality test and Nonparametric test)**


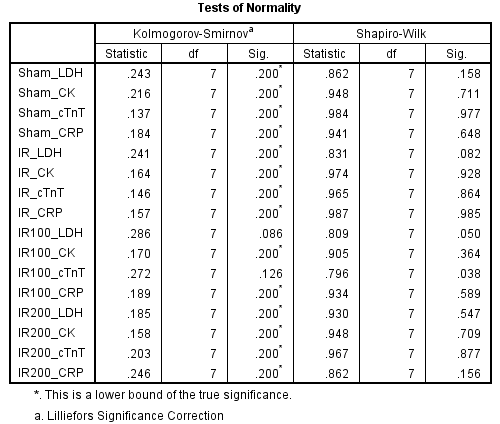


Data less than 0.05 are normally distributed (red box). Nonparametric tests are performed.

**Nonparametric tests (two-independent samples, Mann-Whitney test)**

Treatment groups: 1) Sham group, 2) I/R group, 3) I/R + paeonol 100 mg/kg, I/R + 4) paeonol 200 mg/kg


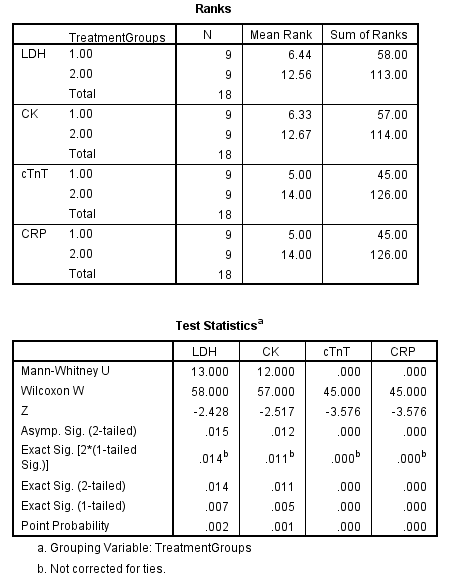


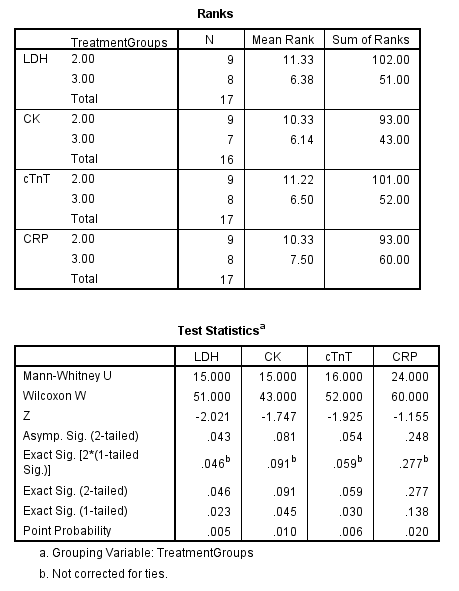


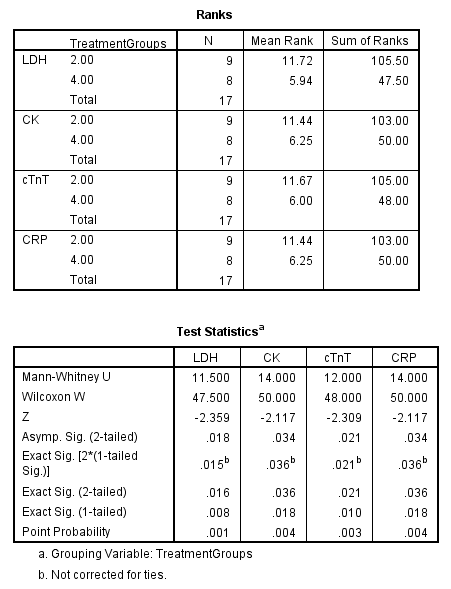


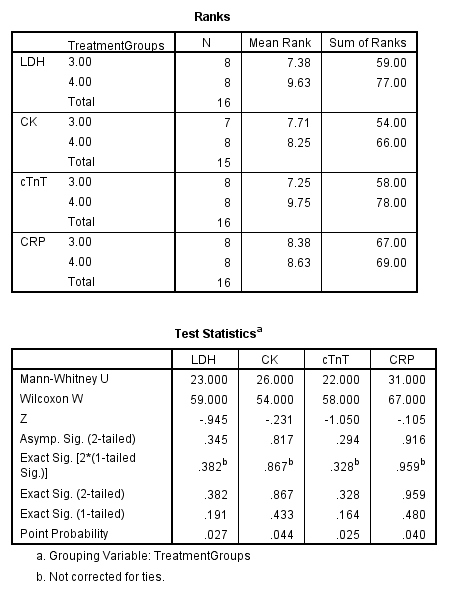


Mean ± SE and SD


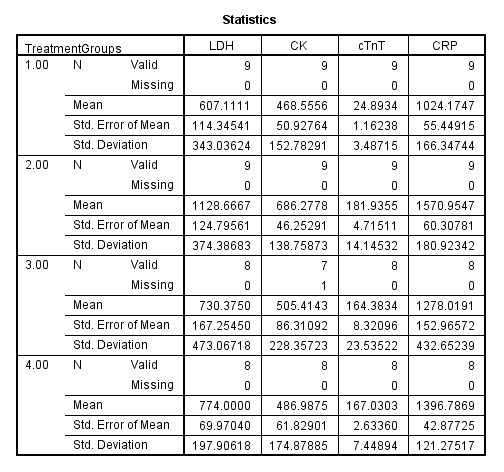

Supplement: Supplementary file 5 [file DataSheet5.DOCX]
